# Is it necessary to screen *Helicobacter pylori* infection in patients with celiac disease and iron deficiency? 

**Published:** 2016

**Authors:** Gabriel Samasca, Diana Deleanu, Genel Sur, Iulia Lupan, Alexandru Giulia, Rahela Carpa

**Affiliations:** 1 *Department of Immunology and Allergology, University of Medicine and Pharmacy, Cluj-Napoca, Romania*; 2 *Department of Pediatrics II, Iuliu Hațieganu, University of Medicine and Pharmacy, Cluj-Napoca, Romania*; 3 *Department of Molecular Biology, “Babes-Bolyai” University, Cluj-Napoca, Romania *; 4 *Department of Microbiology, “Babes-Bolyai” University, Cluj-Napoca, Romania *

**To The Editor**

Early studies have not revealed a link between* Helicobacter pylori *and iron deficiency (1). A recent study from your journal revealed a significant association between *H. pylori *infection and iron deficiency anemia in patients with celiac disease. But the conclusion of this study was that *H. pylori* infection and iron deficiency anemia is poorly reflected in practice to celiac disease patients (2). However, I would like to highlight some points for more clarification of the issue. Long-term clinical outcomes were favorable to *H. pylori *eradication and provided evidence for a cause-effect relation between *H. pylori *and iron deficiency anemia (3). Mild enteropathy was common in patients with iron deficiency anemia and negative celiac disease serology. Final diagnoses in most patients with enteropathy were: gluten sensitive enteropathy with anemia, *H. pylori* infection with anemia, or bouth (4).  *H. pylori *infection was considered a risk factor for anemia due to iron deficiency, mainly in children and adolescent groups with high iron requirements (5). In severe iron deficiency anemia, more than 50% of patients had an active *H. pylori *infection (6). Therefore, we recommend performing the screening for *H. pylori* infection in patients with celiac disease and iron deficiency.
